# Simple Sequence Repeat Marker-Based Genetic Diversity and Chemical Composition Analysis of Ancient *Camellia sinensis* in Jiulong County, Sichuan Province, China

**DOI:** 10.3390/genes15101317

**Published:** 2024-10-14

**Authors:** Haitao Huang, Shuwen He, Xuxia Zheng, Daliang Shi, Peixian Bai, Yun Zhao, Jizhong Yu, Xiaojun Niu

**Affiliations:** 1 Tea Research Institute, Hangzhou Academy of Agricultural Science, Hangzhou 310024, China; hthuang309@hotmail.com (H.H.); xxzheng43@163.com (X.Z.); sdl76984@126.com (D.S.); bpx6535869@126.com (P.B.); yunz2431@163.com (Y.Z.); hchyu@126.com (J.Y.); 2 College of Horticulture, Henan Agricultural University, Zhengzhou 450002, China; 18339208679@163.com

**Keywords:** ancient tea plant, genetic diversity, SSR markers, identification resource

## Abstract

Background/Objectives: The ancient tea plant germplasm resources are rich in genetic diversity and provide an important basis for the genetic diversity in tea germplasm resources. To explore the genetic diversity of ancient tea plant germplasm resources in Jiulong County, Sichuan Province. Methods: 59 ancient tea tree germplasm resources were analyzed using simple sequence repeat (SSR) molecular markers and chemical composition analysis. Results: The results showed that a total of 83 alleles were amplified by 23 pairs of SSR primers, with an average observed allele number (*Na*) of 3.6 and an effective allele number (*Ne*) of 2.335. The average Shannon information index (*I*) and the polymorphic information content (*PIC*) of the primers were 0.896 and 0.446, respectively. The results of the UPGMA cluster analysis showed that 59 ancient tea tree samples could be classified into five different subgroups. Based on the results of chemical composition analysis, two specific tea germplasm resources with high amino acid content, 10 excellent germplasm resources with tea polyphenol content over 20% and some other tea germplasm resources were identified. Conclusions: This study reveals that Jiulong’s ancient tea tree germplasm exhibits significant genetic diversity and includes valuable tea tree planting resources. These findings provide a foundational framework for the conservation, detailed exploration and sustainable utilization of these resources.

## 1. Introduction

The tea plant (*Camellia sinensis* (L.) O. Kuntze) is one of the most important woody economic crops in China, with a cultivation history of more than 3000 years. Ancient tea trees belong to *Sect. Thea* (L.) Dyer and generally refer to tea trees that have been cultivated naturally or artificially for over a hundred years [1,2]. The ancient tea tree has a strong stress resistance and adaptability, while retaining some of the characteristics of primitive tea trees. This means that ancient tea trees may contain many excellent genes, such as high levels of amino acids, tea polyphenols, catechins and some disease, insect, drought and cold resistance genes [3]. Therefore, the identification and evaluation of ancient tea tree germplasm resources are of great value for the origin and domestication, systematic development, genetic analysis, breeding and resource utilization of the tea tree.

Southwest China, including Sichuan Province, is recognized as one of the central areas for the origin of the tea tree [4,5]. However, previous extensive research on ancient tea germplasm resources has mainly focused on Yunnan and Guizhou provinces in China. The tree types of the ancient tea tree germplasm resources in these regions consist mainly of arbors and small arbors and are mainly concentrated in areas with altitudes of 400–2400 m [6,7,8,9,10]. However, shrub-type ancient tea trees and ancient tea trees at altitudes above 2400 m have been the subject of few studies. Sichuan, located in southwest China, is one of the origin points of the tea tree, and its unique geographical location, climatic conditions and ecological environment have nurtured abundant wild and semi-wild tea tree germplasm resources [11]. Jiulong County, Garze Tibetan Autonomous Prefecture is located in the western part of Sichuan Province, with an average altitude of 2000 to 5500 m, and is rich in wildlife resources.

In our previous study, we investigated the distribution area, morphological characteristics and genetic diversity of agronomic traits of Jiulong ancient tea germplasm resources in Garze [12]. We found that the tree type of Jiulong’s ancient tea resources is shrub-type, and there is a rich diversity in various agronomic traits. Among them, 13 are located at an altitude of 2400 m or more and have excellent cold and drought resistance. However, due to a lack of systematic research and protection, some ancient tea tree resources have been at risk of decline or even extinction in recent years. Therefore, it is necessary to further assess the genetic diversity of these ancient tea trees, which can provide some guidelines for future management, protection, favorable gene mining, and utilization of ancient tea tree germplasm resources in the region.

Morphological and molecular markers are two commonly used methods to study the genetic diversity of species and individual phylogenetic relationships. Methods of morphological analysis are usually based on visually observable species characteristics, which have the advantage of being easy to implement in the field but are susceptible to environmental and climatic influences [13]. Molecular markers based on DNA have become the most effective method for variety identification and genetic diversity assessment due to their good stability and resistance to environmental and climatic influences [14]. Among them, SSR (simple sequence repeat) molecular markers have the advantages of abundant quantity, high polymorphism, co-dominant inheritance, good reproducibility, easy detection and low cost [15]. At present, they have been widely used in research on genetic diversity, population genetic structure, germplasm resource identification and other aspects of tea trees and other species [6,16,17,18].

In the present study, the genetic diversity, population structure and physiological and biochemical characteristics of the ancient tea trees were determined by the SSR molecular markers and physicochemical tests. Our aim was to provide a theoretical basis for genetic resource conservation, specific germplasm resource excavation and utilization of ancient tea tree germplasm resources in the future.

## 2. Materials and Methods

### 2.1. Plant Materials

A total of 59 ancient tea plant germplasm resources were used in this study. They were collected from Jiulong County, Garze Tibetan Autonomous Prefecture of Sichuan Province, China. The detailed geographical location information of these ancient tea tree germplasm resources can be found in previous studies [12]. The code and altitude for the ancient tea plant germplasm resources in this study are presented in Table S1. Tender buds from each ancient tea tree germplasm were collected and immediately stored at −20 °C for subsequent DNA extraction. The new shoots with one bud and two leaves were picked and dried using microwave techniques for tea sample fixation [19] and taken back to the laboratory for chemical composition analysis. In addition, the Longjing 43 tea variety grown at the Daqing Valley Base of the Tea Institute of the Hangzhou Academy of Agricultural Sciences was selected as a control variety for chemical composition analysis.

### 2.2. DNA Extraction and Microsatellite Analysis

DNA was extracted using a modified CTAB method [20], followed by quantification of DNA concentration and purity using an ultra-micro-spectrophotometer (N50 Touch, Munich, Germany). Detailed steps for the CTAB modification included a reduction in the centrifugation time of each step to 3 min and drying of DNA in a 65 °C oven. Tender buds from each ancient tea tree germplasm were collected and immediately stored at −20 °C for subsequent DNA extraction. The DNA concentration of each sample was standardized to 20–50 ng/μL before storage at −20 °C. Fifty-two primer sets were selected from the known microsatellite loci in previous references [21,22]. After the primary screening, 23 pairs of primers with good polymorphism and clear electrophoretic bands were selected for subsequent analysis. The primer information was detailed in Table S2.

PCR amplifications comprised 2.0 µL of DNA, 0.5 µL of primer F (10 µmol/L), 0.5 µL of primer R (10 µmol/L), 5.0 µL of Premix PrimeSTAR (containing DNA polymerase, 2× PCR buffer and dNTP mixture) and ddH_2_O added to 10 µL. The PCR reaction procedure was initially denatured at 94 °C for 4 min, followed by 30 cycles of amplification at 94 °C for 30 s, 52–58 °C for 30 s and 72 °C for 45 s, and a final extension at 72 °C for 5 min stored at 4 °C. The SSR-PCR products were separated on an 8% polyacrylamide gel. After electrophoresis, the gel was stained with 0.1% AgNO_3_ and then placed in 2% NaOH color development solution (containing 0.4% 37% formaldehyde) to observe the electrophoretic bands.

### 2.3. Chemical Composition Detection

The total free amino acid content in the tea leaves was determined using the ninhydrin method described by Chen et al. [23]. The total polyphenol content was determined using the Folin–Ciocalteu colorimetric method as described by Teng et al. [24]. Catechin components and caffeine were analyzed via high-performance liquid chromatography (HPLC), following the method outlined by Niu et al. [25]. A calibration curve using known catechin standards was employed to quantify the compounds in tea leaf samples. The experimental data were calculated using three independent replicates.

### 2.4. Data Analysis

The genotype data were obtained by manual reading of the bands, and the detailed method was based on the recording method proposed by Yu et al. [26]. The clear bands within the target fragment range on the electrophoresis map were labeled as A, B, C and D in order of molecular weight. A single band represents a homozygous genotype, and the double bands were heterozygous genotypes, which were recorded as AA, AB, BB, AC, AD, CD, etc. The missing band was recorded as “0”. The original genotype data matrix was built by importing the data into Excel.

GenAlEx 6.5.0.3 software was used to calculate genetic parameters such as allele, observed number of alleles (*Na*), the effective number of alleles (*Ne*), the Shannon diversity information index (*I*), observed heterozygosity (*Ho*) and expected heterozygosity (*He*) [27].

The original genotype data matrix was converted into the format required by the GenAlEx software, and the number of loci, sample size, population size and number of samples in each population were entered in the first row of Excel. The first column in Excel is the sample number. The second column is the population name corresponding to the sample. The third column is the marker column, with each marker occupying 2 columns. The allelic loci are listed, and the genotypes A, B, C, etc. are replaced by numerical forms of 1, 2, 3, etc. Missing loci are represented by ‘0’. Subsequently, based on the method of “Nei. 1983”, the polymorphic information content (*PIC*) of SSR loci and the genetic distance between samples were calculated in PowerMarker v3.25 software [28]. Structure 2.3.3 software was used to analyze the genetic structure of the ancient tea germplasm resources [29]. K-values were set between 2 and 10, and the length of the burn-in period and the number of Markov Chain Monte Carlo (MCMC) operations were set to 100,000 and 500,000, respectively. Each K-value was repeatedly checked 10 times, and the results were exported to the online analysis tool Structure Selector [30] to determine the optimal K value. The FigTree v1.4.4 software was used for cluster analysis following the unweighted average method (UPGMA), which was used to construct the cluster diagram. The significance of the data was analyzed using Student’s *t*-test, and *p*-values less than 0.05 were statistically significant.

## 3. Results

### 3.1. Diversity Analysis of SSR Loci

In this study, 23 pairs of screened SSR primers producing clear bands were used to analyze the 59 samples of Jiulong ancient tea germplasm resources. As shown in Table 1, a total of 83 alleles were amplified by 23 pairs of SSR primers, with an average of 3.6 sites amplified by each primer. The number of alleles that were amplified by the different primers varied greatly. The observed number of alleles (*Na*) ranged from two to nine, with an average effective number of alleles (*Ne*) of 2.335. Eight pairs of primers (TM107, TM157, TM169, TM348, TM376, TM407, TM427 and TM597) amplified more than four alleles, with TM107 having the highest number of alleles amplified at nine. TM364, TM508, TM514 and TM617 had a relatively low number of amplification alleles, with only two alleles in each case. The Shannon information index (*I*) varied between 0.250 and 1.928, with an average of 0.896. The observed heterozygosity (*Ho*) and the expected heterozygosity (*He*) ranged from 0.137 to 0.759 (average 0.461) and from 0.128 to 0.837 (average 0.516), respectively. The polymorphic information content (*PIC*) values ranged from 0.120 to 0.816, with an average of 0.446. *PIC* is an important indicator for the measurement of locus polymorphism [31]. The average *PIC* value in this study ranged from 0.25 to 0.5 level, indicating that the overall genetic diversity of the population was relatively high.

### 3.2. Genetic Structure and UPGMA Cluster Analysis

**Figure 1:**
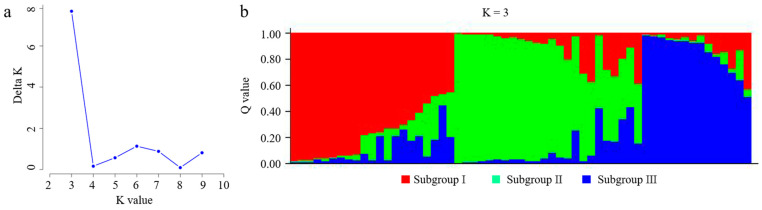
Genetic structure analysis of 59 ancient tea tree samples in Jiulong County. (**a**) Relationship between K and ΔK. (**b**) Model based on population structure analysis of 59 evaluated germplasms (K = 3).

**Figure 2:**
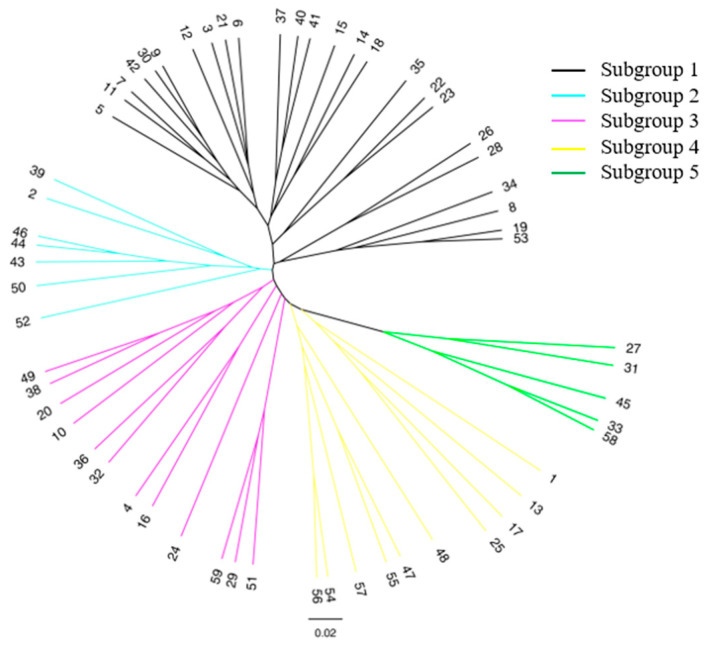
UPGMA evolutionary tree analysis of 59 samples.

The genetic structure of the ancient tea germplasm resource was analyzed using STRUCTURE 2.3.3 software, and the results showed that ΔK was highest at K = 3 (Figure 1a). According to Evanno et al.’s [32] calculation method, 59 materials can be divided into three subgroups (Figure 1b). Among them, subgroup I (red) contains 21 materials, mainly distributed in Kuiduo and Liwu village. Subgroup II (green) contains 24 materials that are mainly distributed in Jianglang, Liwu and Kuiduo villages. Subgroup III (blue) contains 14 materials, distributed mainly in Liwu village. Based on Nei’s genetic distance, the UPGMA clustering method was used to obtain inter-individual clustering maps of the ancient tea germplasm resources. The results show that the 59 ancient tea resources can be divided into five groups (Figure 2). Group 1 includes 21 germplasm resources, mainly distributed in Liwu village and Kuiduo town. Group 2 includes 11 germplasm resources, mainly distributed in Liwu village and Jianglang village of Kuiduo town. Group 3 includes a total of 11 germplasm resources mainly distributed in Liwu village, Jianglang village and Qimulin village in Kuiduo town. Group 4 includes seven germplasm resources mainly distributed in Liwu village and Haidi village of Kuiduo town. Group 5 included nine germplasm resources mainly distributed in Liwu village and Kuiduo village of Kuiduo town. The classification indicated that the clustering analysis of the ancient tea germplasm resources based on the genetic diversity of 23 pairs of SSR markers did not strictly cluster according to altitude and geographical position.

### 3.3. Chemical Composition Analysis

**Figure 3:**
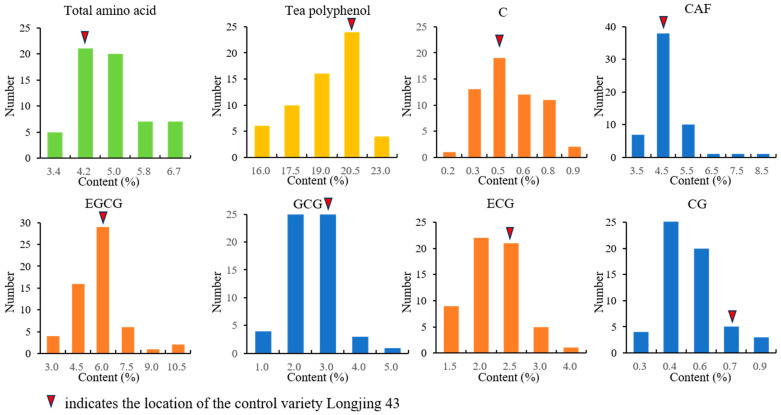
Frequency of distributions of amino acid and catechins in 59 ancient tea resources of Jiulong.

The contents of amino acids, tea polyphenols and catechin components of the ancient tea plants in Jiulong are shown in Figure 3 and Table S3. The total amino acid content was the highest at 6.61% and the lowest at 2.98%, with an average of 4.49%. Among them, the amino acid content of 35 materials was higher than that of the control variety Longjing 43 (4.15%), and that of two materials was higher than 6%. The polyphenol content of the teas ranged from 14.09% to 22.53%, with an average of 18.46%, of which 25 materials had a higher polyphenol content than the Longjing 43 control (19.11%). The caffeine content ranged from 2.71% to 4.94%, with an average 4.02%.

Eight types of catechins were detected. These included four non-ester catechins (GC, EGC, C and EC) and four ester catechins (EGCG, GCG, ECG and CG). The content of GC ranged from 0.32% to 2.12%, with an average of 0.93%. The C content ranged from 0.13% to 0.82%, with an average of 0.43%. The EC content ranged from 0.25% to 1.11%, with an average of 0.63%. The content of EGC ranged from 0.31% to 4.92%, with an average of 1.52%. Two of these materials, Jianglang Village-32 and Kuiduo Village-52, have an EGC content of more than 4%. In terms of ester-type catechins, the EGCG content ranged from 2.10% to 6.73%, the GCG ranged from 0.70% to 2.94%, the ECG ranged from 0.86% to 2.81% and the CG content ranged from 0.17% to 0.82%.

### 3.4. Identification of Special Resources

Based on the “Evaluation Specification for Excellent Crop Germplasm Resources—Tea Tree” (NY/T 2031-2011) compiled by Chen et al. [33], specific and excellent germplasm resources were selected from the tea tree samples of ancient tea trees in Jiulong County. As shown in Table 2, the specific germplasm resources include 14 materials with amino acid content higher than 5%. In two of these materials, Liwu Village Tea Tree Community-3 and Liwu Village Shangshengu-40, the content of amino acid reached 6.1% and 6.6%, respectively. Excellent germplasm resources include 10 materials with tea polyphenol content over 20%, among which the tea polyphenol content of LiWuxianlin-55 in Kuiduo town reaches 22.5%.

In addition, the EGCG and total ester catechin content of the control Longjing 43 were 5.11% and 10.61%, respectively. Among these ancient tea tree germplasm resources, seven materials had an EGCG content of more than 6%, and five materials had a total ester catechin content of more than 11%. The identification and evaluation of these excellent germplasm resources provides a foundation for the utilization of ancient tea germplasm resources.

## 4. Discussion

Genetic diversity plays an important role in plant breeding, sustainable agricultural development and food security [34]. SSR molecular markers have been widely used for the analysis of genetic diversity and the relationships between individuals in populations [35]. In this study, a total of 83 alleles were amplified by 23 pairs of SSR primers, with an average observed allele number (*Na*) of 3.6 and an effective allele number (*Ne*) of 2.335. The observed genetic diversity in Jiulong’s ancient tea germplasm is relatively consistent with earlier studies on tea populations in China (Cai et al. [36]), though notably lower than those reported by Cui et al. [37]. These discrepancies may stem from variations in marker types or differences in population size and geographical distribution. Additionally, the genetic diversity in this region may be influenced by unique environmental pressures or historical cultivation practices. Jiulong County is located in the northern part of the Hengduan Mountains. The highest altitude in the north is 6010 m, and the lowest in the south is only 1440 m, with an average altitude between 2000 and 5500 m. Previous studies have suggested that altitude plays an important role in the genetic diversity of ancient wild tea tree populations [9]. Moreover, Jiulong County also has a long history of tea production, and the famous Tea Horse Ancient Road once passed through here. It is still an important passage between Ganzi Tibetan Autonomous Prefecture and southwestern Sichuan Province. Specific geographical environments and historical cultivation practices may be the reasons for differences in genetic diversity.

Previous studies have shown that the closer the values of *Ho* and *He*, the higher the genetic diversity of the population [38]. The values of *Ho* and *He* in this study were very close, which means that the ancient tea tree germplasm resources of Jiulong had high genetic diversity. In addition, these ancient tea trees have lived in a stable environment for a long time and have not been subjected to strong natural selection or artificial intervention, resulting in a relatively even distribution of gene frequencies within the population and maintaining high genetic stability.

In this study, 59 ancient tea plant germplasm resources were classified into five subgroups using the UPGMA clustering method. Most previous studies have suggested that different tea germplasm resources can be classified mainly on the basis of geographical location [17]. Fang et al. [9] found that altitude may also be an important factor in the clustering the population structure of ancient tea plant germplasm resources. However, the results of the clustering analysis in this study showed that those ancient tea plant germplasm resources did not strictly cluster according to geographical position or altitude. The main reason for this may be that the majority (74.6%) of the ancient tea plants in this study were concentrated distribution in Liwu village, while the number of germplasm resources from other villages was relatively small. In particular, there was only one ancient tea plant in Haidi village with the highest altitude (height: 2808 m). In addition, several villages with ancient tea trees were adjacent to each other, and although there was a significant difference in altitude, the geographical range was relatively narrow, making it difficult to completely separate them geographically. As shown in Figure S1, there are significant differences in the content of amino acids and caffeine between subgroup 1, subgroup 3 and subgroup 4 of the ancient tea tree germplasm resources. Subgroup 3 has the highest amino acid content (average of 4.86%), whereas subgroup 4 has the lowest amino acid content (average of 3.99%). These results may help to better classify, collect and propose some specific strategies to protect the genetic diversity of the ancient tea tree germplasm resources of Jiulong County.

Amino acids are important flavor compounds in tea, and there is often a positive correlation between the level of amino acids and the quality of the tea [39]. Previous studies have confirmed that there are significant differences in the amino acid content of different tea tree varieties, with yellow or white varieties usually having higher amino acid content than green-leaf varieties [40]. In this study, the 59 ancient tea tree germplasm resources were all green varieties, and more than half (59.3%) of the germplasm resources had a higher amino acid content than the control variety Longjing 43. Of these, 14 materials had an amino acid content greater than 5%, and 2 materials had an amino acid content greater than 6%. Considering that green tea tree varieties with a high content of amino acids are relatively rare in the current market, especially in high-altitude areas, the identification and evaluation of these materials can provide theoretical guidance for the collection and protection of germplasm resources in the region, as well as for the backbone parents used to breed excellent varieties in high-altitude areas.

## 5. Conclusions

Ancient tea tree germplasm resources are an important gene pool of tea tree germplasm resources. Exploring their genetic diversity is of great value for the origin and differentiation, systematic development, protection of underutilized resources and development and utilization of tea trees. In this study, 23 pairs of SSR primers with good polymorphism were used to analyze the genetic diversity of 59 ancient tea germplasm resources. The results showed that the ancient tea tree resources in Jiulong have relatively high genetic diversity. The population structure and inter-individual relationship showed that these ancient tea tree resources were not strictly clustered according to altitude and geographical position. In addition, some excellent germplasm resources were identified using chemical composition methods. Based on the results of the molecular cluster analysis combined with the chemical composition analysis, it is more helpful to propose some specific strategies for the protection and utilization of underutilized resources of tea trees in high-altitude areas.

## Figures and Tables

**Table 1:** The genetic parameters of 23 SSR markers.

Loci	No.	*Na*	*Ne*	*I*	*Ho*	*He*	*F*	*PIC*
TM107	57	9	6.130	1.928	0.649	0.837	0.224	0.816
TM157	56	4	1.731	0.704	0.446	0.422	−0.057	0.353
TM169	55	4	3.824	1.362	0.673	0.739	0.089	0.690
TM239	53	3	2.231	0.871	0.453	0.552	0.179	0.450
TM340	52	3	1.491	0.567	0.173	0.329	0.474	0.286
TM348	55	5	1.962	0.870	0.509	0.490	−0.038	0.421
TM364	51	2	1.147	0.250	0.137	0.128	−0.074	0.120
TM369	56	3	1.830	0.704	0.446	0.454	0.016	0.366
TM376	56	8	3.965	1.657	0.464	0.748	0.379	0.717
TM382	58	3	2.204	0.859	0.759	0.546	−0.388	0.443
TM399	58	3	2.691	1.038	0.483	0.628	0.232	0.553
TM406	56	3	1.764	0.736	0.429	0.433	0.010	0.375
TM407	57	4	2.605	1.111	0.526	0.616	0.146	0.554
TM427	59	4	2.063	0.971	0.458	0.515	0.112	0.473
TM493	57	3	2.178	0.855	0.333	0.541	0.384	0.441
TM508	55	2	1.565	0.547	0.327	0.361	0.093	0.296
TM514	57	2	1.761	0.624	0.456	0.432	−0.056	0.339
TM531	59	3	2.283	0.924	0.644	0.562	−0.146	0.481
TM560	55	3	2.303	0.943	0.236	0.566	0.582	0.493
TM564	57	3	2.140	0.820	0.684	0.533	−0.285	0.423
TM586	57	3	2.131	0.818	0.579	0.531	−0.091	0.422
TM597	59	4	1.746	0.758	0.356	0.427	0.167	0.371
TM617	51	2	1.952	0.681	0.373	0.488	0.236	0.369
Mean	56	3.609	2.335	0.896	0.461	0.516	0.095	0.446
SE	0.487	0.360	0.219	0.075	0.033	0.031	0.449	0.031

SE: standard error.

**Table 2:** Excellent tea germplasm resources obtained by evaluating the biochemical components of tea plants.

Germplasm Resource Type	Characteristic	Germplasm Resource Code
Specific resources	Amino acid content ≥ 5%	2~6,9,29,31,36,37,40,53,59,61
Excellent resources	Total catechin content > 20%	17,20,22,30,43,44,45,48,55,58
Others	Total content of ester catechins > 11%	17,39,43,48,53
	EGCG > 6%	16,17,31,39,43,46,53

## Data Availability

The original contributions presented in the study are included in the article; further inquiries can be directed to the corresponding authors.

## Supplementary Materials

The following supporting information can be downloaded at: https://www.mdpi.com/article/10.3390/genes15101317/s1, Table S1: the details information of the 59 samples; Table S2: the primer information of 23 SSR markers; Table S3: the content of amino acids, tea polyphenols and catechins in the 59 germplasm resources of Jiulong ancient tea trees; Figure S1: the content of amino acids (AA), tea polyphenols (TP), caffeine (CAF) and EGCG in different subgroups of ancient tea resources of Jiulong.
