# Supplementary material for: Simple Sequence Repeat Marker-Based Genetic Diversity and Chemical Composition Analysis of Ancient Camellia sinensis in Jiulong County, Sichuan Province, China
Source: Genes (Basel). 2024 Oct 14;15(10):1317. doi: 10.3390/genes15101317 (PMC11507482; doi:10.3390/genes15101317)
Supplement: Supplementary file 1 [file genes-15-01317-s001.zip › genes-3240100-supplementary figure S1.pdf]

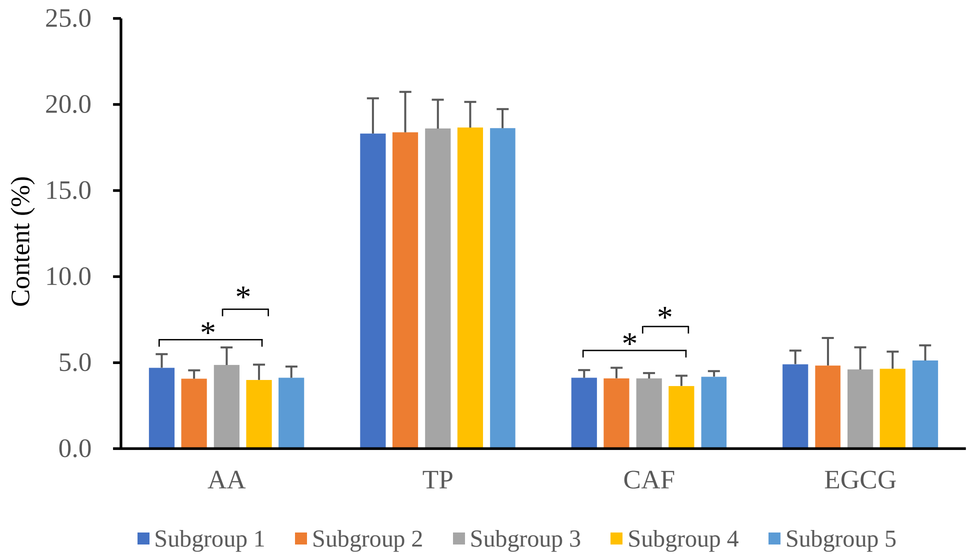

Fig. S1The content of amino acids (AA), tea polyphenols (TP) , caffeine (CAF) , and EGCG in different subgroups of ancient tea resources of Jiulong. * Represents a *P*-value less than 0.05.
